# Seasonality of primary care utilization for respiratory diseases in Ontario: A time-series analysis

**DOI:** 10.1186/1472-6963-8-160

**Published:** 2008-07-28

**Authors:** Rahim Moineddin, Jason X Nie, Gabrielle Domb, Alan M Leong, Ross EG Upshur

**Affiliations:** 1 Department of Family and Community Medicine, University of Toronto, 263 McCaul Street, Toronto, ON, M5T 2W7, Canada; 2 Department of Public Health Sciences, University of Toronto, 6th Floor, Health Sciences Building, 155 College Street, Toronto, ON, M5T 3M7, Canada; 3 Primary Care Research Unit, Sunnybrook Health Sciences Centre, 2075 Bayview Ave, #E-349, Toronto, ON, M4N 3M5, Canada; 4 University of Toronto Joint Centre for Bioethics, 88 College Street, Toronto, Ontario, M5G 1L4, Canada; 5 Institute for Clinical Evaluative Sciences, 2075 Bayview Avenue, Toronto, ON, M4N 3M5, Canada

## Abstract

**Background:**

Respiratory diseases represent a significant burden in primary care. Determining the temporal variation of the overall burden of respiratory diseases on the health care system and their potential causes are keys to understanding disease dynamics in populations and can contribute to the rational management of health care resources.

**Methods:**

A retrospective, cross-sectional time series analysis was used to assess the presence and strength of seasonal and temporal patterns in primary care visits for respiratory diseases in Ontario, Canada, for a 10-year period from January 1, 1992 to December 31, 2002. Data were extracted from the Ontario Health Insurance Plan database for people who had diagnosis codes for chronic obstructive pulmonary disease, asthma, pneumonia, or upper respiratory tract infections.

**Results:**

The results illustrate a clear seasonal pattern in visits to primary care physicians for all respiratory conditions, with a threefold increase in visits during the winter. Age and sex-specific rates show marked increases in visits of young children and in female adults. Multivariate time series methods quantified the interactions among primary care visits, and Granger causality criterion test showed that the respiratory syncytial virus (RSV) and influenza virus influenced asthma (p = 0.0060), COPD (p = 0.0038), pneumonia (p = 0.0001), and respiratory diseases (p = 0.0001).

**Conclusion:**

Primary care visits for respiratory diseases have clear predictable seasonal patterns, driven primarily by viral circulations. Winter visits are threefold higher than summer troughs, indicating a short-term surge on primary health service demands. These findings can aid in effective allocation of resources and services based on seasonal and specific population demands.

## Background

Respiratory diseases such as asthma, pneumonia, and chronic obstructive pulmonary disease (COPD) rank among the leading causes of death in Canada and abroad [1,2]. The burden of respiratory diseases in primary care is significant. In 2001/2002, respiratory disorders were among the most common reason for office visits to family physicians, with a large portion of the total visit volume due to upper respiratory tract infections, followed by asthma, pneumonia, and COPD [3]. Two of the more common causes of substantial respiratory morbidity and mortality are respiratory syncytial virus (RSV) and influenza virus, which are known to interact, and co-circulate in the Province of Ontario [4].

The impact of respiratory diseases affirms the need to have a more in-depth understanding of the effects in health service utilization. A previous study of health services utilization examined the impact of respiratory viruses on hospital admissions [5]. The general aim of this study is to extend the methodology to the primary care context. The specific objectives of this study are: 1) to examine temporal trends and assess the strength of seasonal patterns for respiratory infections, asthma, pneumonia, and COPD on primary care visits, by age and sex using Fisher Kappa and Bartlett-Kolmogorov-Smirnov tests, 2) to examine the interactions in primary care visits of respiratory conditions by using multivariate time-series methods, and 3) using the Granger causality criterion, to test the effects of respiratory syncytial virus (RSV) and influenza virus on primary care visits for respiratory disease.

## Methods

We conducted a retrospective, population-based study to assess temporal patterns and interactions in primary care visits for respiratory diseases in Ontario, Canada, for a 10-year period from January 1, 1992 to December 31, 2002. Data were extracted from the Ontario Health Insurance Plan (OHIP) database for people who had diagnosis codes for one of four respiratory disease groups: COPD; asthma; pneumonia, or upper respiratory tract infections. The following ICD-9 classification codes were used to determine disease groupings: COPD (491, 492, and 496), asthma (493), pneumonia (486), and upper respiratory tract infections (487, 460, and 466). All primary care physician visits for each disease were extracted. Weekly isolates of viral subtypes (RSV and influenza) covering the study period were obtained from Health Canada.

Patients in each diagnostic group were included in the data analysis if they had made at least one visit to a physician for which the diagnostic code corresponded to that disease. Visits were restricted to those made to a general practitioner (GP) or family practitioner (FP) for primary care in the office, home, or long-term care facility. Emergency department and inpatient visits were excluded from the calculation. Visit rates to primary care physicians per 100,000 persons were calculated using the population of Ontario for the study period. This was obtained using annual census data provided by Statistics Canada and normalized for length of month. Monthly and weekly population estimates were derived through linear interpolation. All transfers from one acute care hospital to another within this study group were excluded from the dataset.

### Statistical methods

The rates of primary care visits for respiratory diseases were log transformed in order to stabilize the variance. Visual inspection of the time plots and the autocorrelation functions for the log transformed rates showed clear seasonality of period 52 in all four time series. Spectral analysis was conducted to assess cyclical patterns in the rates of primary care visits. The Fisher-Kappa (FK) test was used to detect a major sinusoidal component buried in white noise and the Bartlett-Kolmogorov Smirnov (BKS) test was used to detect departure from the white noise hypothesis over all frequencies. Both FK and BKS tests were strongly significant (p < 0.0001) confirming seasonality of the series. The seasonality components were eliminated by differencing of order 52. That is, if *x*_*t *_shows the value of a series at time *t*, then the seasonal differencing of order 52 is *x*_*t *_- *x*_*t*-52_. Visual inspection of the differenced series confirmed stationarity.

In practice, much of time-series data consists of measuring several continuous variables simultaneously over time, which best can be considered as components of a vector-valued (multivariate) time series. When the components of a vector-valued time series are related to each other, then the interdependence between series must be taken into account in the analysis. The extension of univariate time-series models to multivariate models allows researchers to analyze interdependent time-series data properly. Let us consider an autoregressive model of order 1 $\left(\begin{array}{c} X_{1t}\\ X_{2t} \end{array}\right)=\left(\begin{array}{c} \varphi_{10}\\ \varphi_{20} \end{array}\right)+\left[\begin{array}{cc} \varphi_{11} & \varphi_{12}\\ \varphi_{21} & \varphi_{22} \end{array}\right]\left(\begin{array}{c} X_{1t-1}\\ X_{2t-1} \end{array}\right)+\left(\begin{array}{c} e_{1t}\\ e_{2t} \end{array}\right)$ where ${\left(\begin{array}{cc} X_{1t} & X_{2t} \end{array}\right)}^{\prime }$ is a 2-dimensional time series vector of random variables of interest, $\left(\begin{array}{cc} e_{1t} & e_{2t} \end{array}\right)$ is a vector white noise process with mean zero and variance covariance matrix Σ, $\left(\begin{array}{c} \varphi_{10}\\ \varphi_{20} \end{array}\right)$ and $\left[\begin{array}{cc} \varphi_{11} & \varphi_{12}\\ \varphi_{21} & \varphi_{22} \end{array}\right]$ are matrices of unknown parameters. Expansion of the above multivariate autoregressive model provides two univariate equations *X*_1*t *_= *φ*_10 _+ *φ*_11_*X*_1*t*-1 _+ *φ*_12_*X*_2*t*-1 _+ *e*_1*t *_and *X*_2*t *_= *φ*_20 _+ *φ*_21_*X*_1*t*-1 _+ *φ*_22_*X*_2*t*-1 _+ *e*_2*t*_. The parameters of multivariate autoregressive model have important interpretations. The main diagonal elements of the estimated matrix parameter of the multivariate autoregressive model (*φ*_11_, *φ*_22_) show the temporal contribution of the past values on the present value. The off-diagonal elements (*φ*_12_, *φ*_21_) show the contribution of the past values of the other series on the present value of that series. When there is no feedback relationship, the off-diagonal elements of the coefficient matrices are statistically zero [6].

In econometrics, the Granger Causality Test [7,8] has been used extensively for determining the Granger causal relationship among series. Suppose two time series {*x*_*t*_} and {*y*_*t*_} are observed and we are interested to know whether movements in {*x*_*t*_} precede movements in {*y*_*t*_} or is it the opposite, or are the movements contemporaneous? In other words, the variable *x*_*t *_is causing *y*_*t *_if we can predict *y*_*t *_better by using all available information, and *x*_*t *_than if all the information apart from *x*_*t *_had been used.

The multivariate time-series model is fitted to data using the VARMAX procedure of the SAS 9.1 statistical package (SAS Institute Inc., Cary, NC, USA). Ethical approval for this study was obtained from the Sunnybrook Health Sciences Centre Research Ethics Board.

## Results

### Overall trend and seasonality

**Figure 1:**
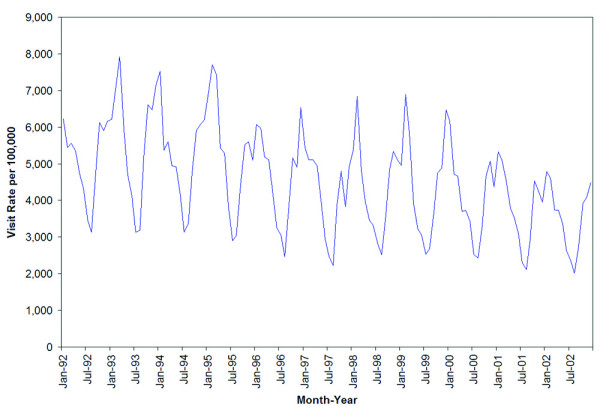
Overall rates of respiratory visits to primary care providers, by month and year, in Ontario, 1992–2002.

The overall respiratory primary care visit rates show clear seasonal variation (Figure 1). Over the study period from January 1992 through December 2002, peaks in incidence occur annually from the winter (December, January, and February) to early spring (March and April), corresponding to influenza and RSV circulations. Visits peak biannually during spring and fall. The magnitude of each cycle declined over the study period, suggesting a stabilization of visits to primary care providers. Winter visits are threefold higher than summer (June, July, and August) troughs, which indicate an important short-term surge on health service demand. As evident from Figure 1, the overall primary care visit rates for respiratory diseases also declined over the study period (beta = -15.5, p < 0.0001).

**Figure 2:**
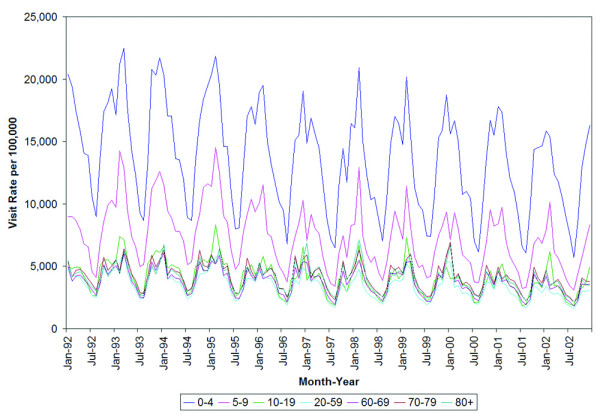
Rates of respiratory visits to primary care providers by month and age group.

Seasonal variation is evident across all age groups (Figure 2). Children from birth to age 4 were the most frequent group to visit primary care providers for respiratory disease conditions, followed by children aged 5 to 9 and 10 to 19, respectively. These three groups exhibited the greatest seasonal variation, evident in large fluctuations, by a magnitude of approximately 12,000 visits in the youngest age group. There was comparatively less variation in visit rates among adults aged 20 and older, who also had the lowest overall visit rates among all age groups.

**Figure 3:**
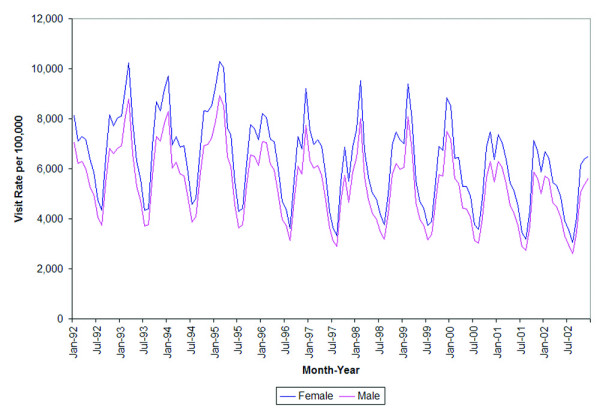
Rates of respiratory visits to primary care providers by month and gender.

Seasonal variation in both genders is evident and nearly identical. Females paid significantly more visits to primary care providers for respiratory disease than males (p < 0.0001) throughout the study period (Figure 3). This magnitude of difference between the two groups amounts to roughly 500 visits per 100,000 people.

### Trend and seasonality by specific disease condition

**Figure 4:**
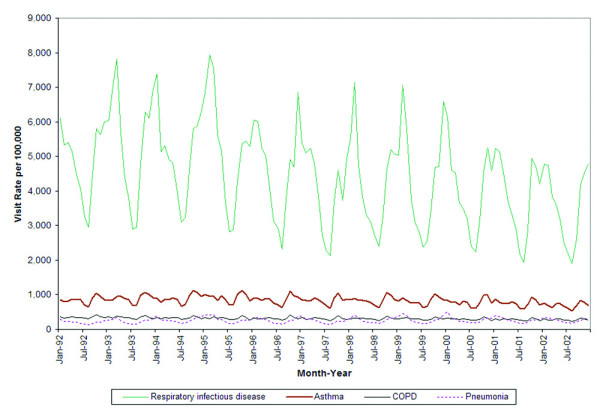
Rates of respiratory visits to primary care providers by month and disease condition.

Respiratory infectious diseases accounted for a total of 66,005,991 primary care visits over the 11-year period, followed by a total of 12,331,563 visits for asthma, 4,662,735 visits for COPD, and 3,846,481 visits for pneumonia. The overall primary care visit rates for each of the four diseases exhibited a significant downward trend over the 10 year period (COPD: beta = -0.5, p < 0.0001, Asthma: beta = -1.4, p < 0.0001 pneumonia: beta = 0.3, p = 0.0004, respiratory infections: beta = -13.8, p < 0.0001). Clear and statistically significant seasonal patterns for the overall population were observed for respiratory infectious diseases, asthma, COPD, and pneumonia (Figure 4). The R^2^_autoreg _of all ages and both genders for asthma, COPD, pneumonia, and respiratory infections were all above 0.8, representing a very strong seasonal effect [9].

### Multivariate models

Let the vector (*a*_*t*_, *c*_*t*_, *p*_*t*_, *r*_*t*_) represent the log transformed and seasonally differenced rates of asthma, COPD, pneumonia, and upper respiratory tract infections per 100,000 populations respectively, at time *t*. In calculating the rate for each series, the numerator consisted of the number of diseases for each week, and the denominators were constructed from annual census data provided by Statistics Canada for the residents of the province of Ontario. From the data, weekly visit rates per 100,000 populations were calculated for each disease. Visual inspection of the time plots of the seasonally differenced rates of diseases did not reveal any nonstationarity in the series. A vector autoregressive model of order 3 was fitted, and all statistically significant elements of the estimated coefficients matrices were inspected. The estimated model (p-values are in brackets) is as follows:

$$\begin{array}{l} \left(\begin{array}{c} a_{t}\\ c_{t}\\ p_{t}\\ r_{t} \end{array}\right)=\left(\begin{array}{c} \underset{\left(0.0001\right)}{-0.02}\\ \underset{\left(0.0001\right)}{-0.02}\\ \underset{\left(0.0050\right)}{-0.01}\\ \underset{\left(0.0001\right)}{-0.02} \end{array}\right)+\left(\begin{array}{cccc} \underset{\left(0.0001\right)}{0.42} & \underset{\left(0.0001\right)}{-0.47} & \underset{\left(0.7773\right)}{0.02} & \underset{\left(0.0018\right)}{0.21}\\ \underset{\left(0.0360\right)}{-0.21} & \underset{\left(0.2378\right)}{0.10} & \underset{\left(0.1557\right)}{0.13} & \underset{\left(0.1061\right)}{0.11}\\ \underset{\left(0.0002\right)}{-0.32} & \underset{\left(0.0001\right)}{-0.51} & \underset{\left(0.0001\right)}{1.03} & \underset{\left(0.0001\right)}{0.39}\\ \underset{\left(0.0334\right)}{0.23} & \underset{\left(0.0001\right)}{-0.64} & \underset{\left(0.0020\right)}{0.31} & \underset{\left(0.0001\right)}{1.03} \end{array}\right)\left(\begin{array}{c} a_{t-1}\\ c_{t-1}\\ p_{t-1}\\ r_{t-1} \end{array}\right)+\left(\begin{array}{cccc} \underset{\text{(0}\text{.6584)}}{\text{0}\text{.05}} & \underset{\left(0.8768\right)}{\text{-0}\text{.01}} & \underset{\text{(0}\text{.0868)}}{\text{0}\text{.20}} & \underset{\text{(0}\text{.8003)}}{\text{-0}\text{.02}}\\ \underset{\text{(0}\text{.5807)}}{\text{-0}\text{.06}} & \underset{\text{(0}\text{.4716)}}{\text{0}\text{.07}} & \underset{\left(0.4147\right)}{0.10} & \underset{\left(0.5274\right)}{0.06}\\ \underset{\left(0.7812\right)}{0.03} & \underset{\left(0.2394\right)}{0.10} & \underset{\left(0.5773\right)}{0.06} & \underset{\left(0.1954\right)}{-0.10}\\ \underset{\left(0.6619\right)}{-0.06} & \underset{\left(0.0664\right)}{0.21} & \underset{\left(0.7178\right)}{-0.05} & \underset{\left(0.8916\right)}{0.01} \end{array}\right)\left(\begin{array}{c} a_{t-2}\\ c_{t-2}\\ p_{t-2}\\ r_{t-2} \end{array}\right)\\ \;+\left(\begin{array}{cccc} \underset{\text{(0}\text{.0041)}}{\text{0}\text{.26}} & \underset{\text{(0}\text{.4343)}}{\text{0}\text{.06}} & \underset{\text{(0}\text{.3147)}}{\text{-0}\text{.08}} & \underset{\text{(0}\text{.0001)}}{\text{-0}\text{.26}}\\ \underset{\text{(0}\text{.2220)}}{\text{0}\text{.12}} & \underset{\text{(0}\text{.0983)}}{\text{0}\text{.15}} & \underset{\left(0.1541\right)}{-0.12} & \underset{\left(0.0427\right)}{-0.14}\\ \underset{\left(0.0003\right)}{0.31} & \underset{\left(0.8282\right)}{-0.02} & \underset{\left(0.1729\right)}{-0.10} & \underset{\left(0.0001\right)}{-0.32}\\ \underset{\left(0.0004\right)}{0.38} & \underset{\left(0.8372\right)}{-0.02} & \underset{\left(0.0829\right)}{-0.16} & \underset{\left(0.0001\right)}{-0.31} \end{array}\right)\left(\begin{array}{c} a_{t-3}\\ c_{t-3}\\ p_{t-3}\\ r_{t-3} \end{array}\right) \end{array}$$

The Portmanteau test for cross correlation of residuals, Durbin Watson tests, and the univariate model AR diagnostics were non-significant, while the R-squared for each series was highly significant (ranged from 0.44 to 0.87, p < 0.0001). These diagnostic tests for residuals showed acceptable model fit. (The non-significant estimates (p > 0.01) are not presented in the following equations.) Expansion of the above multivariate autoregressive model provided four univariate equations which represent the functional relationships between each series and the others. These four equations are

$$\begin{array}{l} a_{t}=-0.02+0.42a_{t-1}-0.47c_{t-1}+0.21r_{t-1}+0.26a_{t-3}-0.26r_{t-3}\\ c_{t}=-0.02-0.21a_{t-1}-0.14r_{t-3}\\ p_{t}=-0.01-0.32a_{t-1}-0.51c_{t-1}+1.03p_{t-1}+0.39r_{t-1}+0.31a_{t-3}-0.32r_{t-3}\\ r_{t}=-0.02-0.23a_{t-1}-0.64c_{t-1}+0.31p_{t-1}+1.03r_{t-1}+0.38a_{t-3}-0.31r_{t-3} \end{array}$$

The first equation shows that the rate of asthma at time *t *is negatively affected by the previous week's the rate of COPD, but positively affected by the rate of respiratory diseases. The rate of COPD at time *t *is negatively affected by previous week's rate of asthma. This consistent feedback relationship between Asthma and COPD reflect the difficulty of separating the visits for these two conditions. Asthma and COPD had negative effects on pneumonia, while respiratory diseases had a positive effect. That is if rate of respiratory visits increases this week then rate of pneumonia will increase next week. Similarly if rates of COPD or asthma increase this week then rate of pneumonia will decrease next week. Lastly, respiratory diseases were negatively affected by previous week's rate of COPD and positively affected by pneumonia. The Granger Causality Test showed that influenza and RSV cause asthma, COPD, pneumonia, and respiratory diseases, *p*-values are 0.0060, 0.0038, 0.0001, and 0.0001 respectively.

## Discussion

This study presents several important findings regarding seasonal patterns, and interactions of respiratory diseases on total primary care visit rates, in Ontario over the 10 year study period. A clear seasonal variation exists with peaks in the winter months and troughs in the summer. Consistent with other studies, the highest rates were found in the younger age groups (ages 0 to 4 and 5 to 9) [10], and females were found to be a greater driving factor in higher overall visit rates.

The seasonal pattern of influenza and pneumonia can be primarily attributed to viral infections that are caused by influenza and RSV circulations during the winter months [5]. Other postulated factors including temperature, the crowding together of people indoors during the winter, and the increased contact among children in the school environment following summer holidays, are less compelling contributors as the onset of peak viral activity varies from season to season and the variation in peaks does not correlate with temperature or school attendance [11,12]. Adverse air quality is predominantly an issue in the summer months in Ontario, thus it plays a considerably lesser role in respiratory morbidity at the population level than the presence of respiratory viruses.

The seasonal variability and general trend of overall primary care visit rates are predominately driven by the high rates among the younger age groups (0–4 years, 5–9 years). Respiratory disease is the leading cause of morbidity in children of developed countries [13]. Viral infections, such as those caused by RSV, predominantly affect infants and very young children, in the form of bronchiolitis and pneumonia. This, along with the increased susceptibility to environmental exposure of young children, can be explained by an underdeveloped immune system [11]. A strength of this research is the analysis of seasonal patterns across narrowly-defined age-groups in the younger population, who are more susceptible to these respiratory conditions. The difference in primary care visit rates by gender may be due to several factors. Higher overall visit rates among females have been attributed to sex differences in lung characteristics [14]. Although data is not shown, results by age group show that males have a higher rate of visits from birth to age 4. However, there is a significant increase in female visits in the 10 to 19 age group and older. Various studies have attributed the timing of this difference to sex hormones [13,15].

There are significant interactions among respiratory diseases for primary care visits. The effect of the past values of some conditions on the present rates of other conditions probably reflect the difficulty of separating the visits for these clinical conditions, and hence one can often masquerade as the other. However, there is some plausibility for more serious invasive diseases like pneumonia to peak after the onset of acute respiratory diseases reflecting a longer time course in populations required for invasive disease. The decline in pneumonia diagnosis subsequent to COPD and asthma peaks is a novel finding, and again likely reflects the fact that these conditions can present similarly. Coding practices may also play a role. Further investigation is warranted. Improving the linkage of viral isolate data and primary care visits would strengthen future epidemiological analysis, with the more refined and nuanced linkage of exposure to outcome and also making possible predictive modeling.

Limitations in this study included the use of administrative data to determine number of primary care visits, which were not originally collected with the intention for use in health research. The diagnostic codes cited in physician claims have not been validated and may be unreliable. Patients included in data analysis may have been included with only uncertain diagnoses, which could have been subsequently ruled out by test results or further examination. Further, physicians assigned only one diagnosis per patient visit, which meant that secondary or other subsequent diagnoses were excluded. Moreover, bronchitis and COPD are difficult to distinguish by physicians, and may lead to an underestimation of COPD diagnoses [16]. Therefore, the results presented may have underestimated primary care utilization for respiratory diseases in Ontario.

The decline in visits over the time period is unexplained. Further research to determine whether this is a result of billing practices, the introduction of primary care reform with new models of funding, or the impact of a universal influenza immunization program introduced in Ontario in 2000 is required.

## Conclusion

Respiratory diseases are, for the most part, preventable health conditions, with clear predictable seasonal patterns driven primarily by circulating viral pathogens. The visit rates to primary care providers are substantial and directly impact planning of health services. The results of this study support the need to introduce seasonal programs to reduce the impact of respiratory conditions. Furthermore, winter visits are threefold higher than summer troughs, which indicate an important short-term surge on primary health service demands. These findings can aid in effective allocation of resources and services based on seasonal and specific population demands.

## Competing interests

The authors declare they have no competing interests.

## Authors' contributions

All authors contributed to every stage of the manuscript from its conceptualization to the revision of the final draft.

## Pre-publication history

The pre-publication history for this paper can be accessed here:


